# Supplementary material for: WordCommentsAnalyzer: A windows software tool for qualitative research
Source: F1000Res. 2018 Sep 4;7:536. Originally published 2018 May 3. [Version 2] doi: 10.12688/f1000research.14819.2 (PMC6137411; doi:10.12688/f1000research.14819.2)
Supplement: Supplementary file 3 [file f1000research-7-17614-s0002.tgz › b2a79496-4ada-47c4-9682-a99ce867836d.zip!/Eight Iranian Health Journals/Journal of Research in Rehabilitation Sciences _6.docx]

© Journal of Research in Rehabilitation Sciences . This work is licensed under CC BY-NC 4.0 (https://creativecommons.org/licenses/by-nc/4.0/).

Volume & Issue: دوره 12، شماره 5: 1395

Title: ارتباط تاب‌آوری با بار مراقبتی والدین دانش‌‌آموزان کم‌توان ذهنی استان گلستان در سال 1395

http://jrrs.mui.ac.ir/index.php/jrrs/article/view/2752

مقدمه: وجود کودک کم‌توان ذهنی، گاهی بر هم‌ زننده سلامت روانی خانواده است و سبب آسیب جدی به والدین می‌گردد. بررسی و توجه به وضعیت سلامت روانی والدین کودکان استثنایی با توجه به بار‌های روانی، اجتماعی و مالی ناشی از مراقبت و افزایش بار مراقبتی در خانواده‌های دارای کودک کم‌توان ذهنی، ضروری به نظر می‌رسد. مواد و روش‌ها: مطالعه حاضر از نوع توصیفی- همبستگی بود. نمونه‌های پژوهش را 200 نفر از والدین دانش‌آموزان مشغول به تحصیل در مدارس استثنایی استان گلستان در سال 1395 تشکیل داد. نمونه‌ها با روش نمونه‌گیری تصادفی چند مرحله‌ای انتخاب ‌شدند. جهت جمع‌آوری داده‌ها از پرسش‌نامه اطلاعات دموگرافیک، مقیاس تاب‌آوری Conner-Davidson (Connor-Davidson Resilience Scale یا CD-RISC) و مقياس بار مراقبتی Elmstahl (Caregiver Burden Scale) استفاده گردید. داده‌های به دست آمده با استفاده از آمار توصیفی و استنباطي در نرم‌افزار SPSS و در سطح معنی‌داری 05/0 > P مورد تجزیه ‌و تحلیل قرار گرفت. یافته‌ها: تاب‌آوری والدین کودکان کم‌توان ذهنی، 27/17 ± 86/51 و بار مراقبتی آنان 27/14 ± 89/56 بود. بر اساس آزمون رگرسیون خطی ساده، بین تاب‌آوری و بار مراقبتی ارتباط معنی‌داری مشاهده شد [(1/8- تا 2/4-) 6- = Confidence interval (CI) 95 درصد، 010/0 > P، 39/0 = Beta، 32/0 = B])؛ به‌ طوری‌ که به‌ ازای هر یک درجه کاهش تاب‌آوری، میزان بار مراقبتی 3/0 نمره بیشتر شد. نتیجه‎گیری: در مطالعه حاضر، میزان بار مراقبتی بالاتر و تاب‌آوری پایین‌تر از حد متوسط بود و با کاهش میزان بار مراقبتی، می‌توان تاب‌آوری والدین دانش‌آموزان کم‌توان ذهنی را افزایش داد. از میان متغیرهای پیشگویی‌ کننده، تأثیر میزان درامد بر بار مراقبتی از سایر متغیرها در والدین دارای کودک کم‌توان ذهنی به میزان بیشتری مشهود بود که این امر نشان دهنده این است که با بالا بردن سطح درامد و رفاه اقتصادی، می‌توان بار مراقبتی را کاهش و تاب‌آوری را افزایش داد. بنابراین، پیشنهاد می‌شود که تمهیدات اقتصادی و مشاوره لازم در جهت کاهش میزان بار مراقبتی صورت گیرد.

Title: اثر هشت هفته تمرينات پيلاتس بر برخي شاخص‌هاي آمادگي جسماني و عملکرد حرکتي دانش‌‌آموزان دختر کم‌توان ذهني

http://jrrs.mui.ac.ir/index.php/jrrs/article/view/2747

مقدمه: انجام تمرینات پيلاتس در افراد سالم باعث افزايش استقامت قلبي- تنفسي، عضلاني و انعطاف‌پذيري می‌شود که به این نکته در پژوهش‌های مختلف اشاره ‌شده است. هدف از انجام مطالعه حاضر، تعيين اثر تمرين پيلاتس بر برخي شاخص‌های آمادگي جسماني و عملکرد حرکتي دانش‌آموزان دختر کم‌توان ذهني بود. مواد و روش‌ها: اين تحقیق به صورت تجربي بر روي 40 آزمودني کم‌توان ذهنی که به ‌طور داوطلبانه و در دسترس در پژوهش شرکت کردند، انجام شد. آزمودنی‌ها به طور تصادفي در دو گروه 20 نفره شاهد و تجربی قرار گرفتند. نمونه‌های گروه آزمايش تمرينات پيلاتس را به مدت هشت هفته 3 جلسه‌ای یک ‌ساعته انجام دادند و گروه شاهد در طول پژوهش در هیچ برنامه منظم ورزشی شرکت نکردند و در طول انجام مطالعه به زندگی طبیعی خود ادامه دادند. 24 ساعت قبل و بعد از برنامه تمريني، استقامت قلبي- تنفسي، قدرت عضلاني، استقامت عضلاني، انعطاف‌پذيري، شاخص‌هاي ترکيب بدن [(قد، وزن، درصد چربي بدن، شاخص توده بدني (Body mass index یا BMI)، نسبت دور کمر به دور باسن (Waist to Hip Ratio یا WHR)] و عملکرد حرکتی اندازه‌گيري شد. جهت مقايسه تغييرات درون گروهي و ميانگين داده‌هاي جمع‌آوری ‌شده از آزمون Paired t استفاده گردید. یافته‌ها: در گروه شاهد بين ميانگين‌هاي پيش‌آزمون و پس‌آزمون، در هيچ يک از متغيرها تفاوت معنی‌داري مشاهده نشد، اما تمرینات پیلاتس در گروه تجربی بر روی همه متغیرهای دانش‌آموزان دختر کم‌توان ذهني تأثير معنی‌داري داشت. نتیجه‎گیری: به نظر مي‌رسد که انجام هشت هفته تمرين پيلاتس، باعث ارتقای شاخص‌های آمادگي جسماني دانش‌آموزان دختر کم‌توان ذهني و پيشرفت در عملکرد حرکتي آن‌ها شد و در نتیجه، بهبود کیفیت زندگی کودکان کم‌توان ذهني را به دنبال داشت.

Title: کینماتیک اندام تحتانی حین راه رفتن در افراد دارای ناهنجاری هایپرلوردوزیس کمری

http://jrrs.mui.ac.ir/index.php/jrrs/article/view/2744

مقدمه: راه رفتن به‌ عنوان یک فعالیت عضلانی مستمر، تأثیر فراوانی در شکل‌گیری وضعیت بدنی افراد دارد و وضعیت بدنی افراد نیز به‌گونه‌ای متقابل، می‌تواند راه رفتن آنان را تحت تأثیر قرار دهد. مطالعه حاضر با هدف مقایسه ویژگی‌های کینماتیکی اندام تحتانی حین راه رفتن در افراد دچار ناهنجاری هایپرلوردوزیس کمری با افراد سالم انجام شد. روش: این تحقیق از نوع توصیفی- مقایسه‌ای بود و در آن 38 نفر از دانشجویان دختر به‌ روش نمونه‌گیری هدفمند و در دسترس انتخاب شدند و در دو گروه سالم و هایپرلوردوزیس قرار گرفتند. زاویه لوردوز کمری با استفاده از خط‌کش منعطف و اطلاعات راه رفتن آزمودنی‌ها با استفاده از سیستم اپتوالکتریک سه ‌بعدی Motion Analysis به وسیله شش دوربین اندازه‌گیری شد. داده‌های مطالعه با استفاده از آزمون‌های Independent t و تحلیل کوواریانس در سطح معنی‌داری 05/0 ≥ P مورد تجزیه و تحلیل قرار گرفت. یافته‌ها: جابه‌جایی زاویه‌ای و سرعت زاویه‌ای مفصل ران و مچ پا و همچنین، زاویه والگوس مفصل زانو در فاز استانس راه رفتن در افراد دارای ناهنجاری هایپرلوردوزیس کمری نسبت به افراد سالم افزایش معنی‌داری را نشان داد، اما تغییری بین جابه‌جایی زاویه‌ای و سرعت زاویه‌ای مفصل زانو در فاز استانس راه رفتن دو گروه مشاهده نشد. نتیجه‌گیری: ناهنجاری لوردوز کمری در صفحه ساجیتال، موجب تغییر در دامنه حرکتی مفصل مچ پا و ران و در صفحه فرونتال، موجب تغییر در زاویه والگوس زانو می‌شود.

Title: مقایسه فعالیت عصبی- عضلانی و زمان‌بندی عضلات منتخب اندام تحتانی در ورزشکاران مبتلا به ژنوواروم و طبیعی حین دویدن: مطالعه مورد- شاهدی

http://jrrs.mui.ac.ir/index.php/jrrs/article/view/2754

مقدمه: راستاي نامناسب اندام تحتاني به ‌ویژه زانو، به دليل تأثير بر عملكرد عضلات، می‌تواند منجر به وقوع آسيب شود. از این‌رو، هدف از انجام مطالعه حاضر، مقایسه فعالیت عصبی- عضلانی و زمان‌بندی عضلات منتخب اندام تحتانی در ورزشکاران مبتلابه ژنوواروم و طبیعی حین دویدن بود. مواد و روش‌ها: در این مطالعه نیمه تجربی، 30 ورزشکار مرد با دامنه سنی 20 تا 25 سال در دو گروه ژنوواروم و طبیعی مورد بررسی قرار گرفتند. با استفاده از اطلاعات حاصل از دستگاه الکترومایوگرافی (Electromyography یا EMG)، میزان و زمان شروع فعالیت عضلات محاسبه شد. جهت بررسی اختلافات بین گروهی از آزمون MANOVA و برای بررسی اختلافات درون‌ گروهی از آزمون Paired t در سطح معنی‌داری 05/0 > P استفاده گردید. داده‌ها در نرم‌افزار MATLAB و SPSS مورد تجزیه ‌و تحلیل قرار گرفت. یافته‌ها: عضله گلوتئوس مدیوس در گروه ورزشکاران دارای ژنوواروم به‌ طور معنی‌داری میزان فعالیت بیشتری نسبت به گروه طبیعی در اندام برتر (032/0 = P) و غیر برتر (039/0 = P) داشت، اما میزان فعالیت عضلات رکتوس فموریس و گاستروکنمیوس داخلی بین دو گروه تفاوت معنی‌داری را نشان نداد (050/0 < P). همچنین، بین زمان شروع فعالیت عضلات اختلاف معنی‌داری مشاهده نشد (050/0 < P). در میزان و زمان شروع فعالیت عضلات بین اندام‌های برتر و غیر برتر تفاوت معنی‌داری وجود نداشت (050/0 < P). نتیجه‎گیری: نتایج مطالعه حاضر نشان داد که افراد داراي بدشکلی ژنوواروم، فعالیت بیشتری در عضله گلوتئوس مدیوس نسبت به افراد طبیعی دارند و این افزایش فعالیت می‌تواند با افزایش نیروهای فشاری و بارهای مفصلی همراه باشد که در طولانی ‌مدت باعث بروز بیماری‌های تخریب مفصلی مانند استئوآرتریت مفصل ران می‌شود.

Title: تمرین با چالش ناپایداری تکلیف- ویژه در عملکرد و اکتساب یک مهارت پیچیده حرکتی

http://jrrs.mui.ac.ir/index.php/jrrs/article/view/2769

مقدمه: مطالعات اکتساب مهارت حرکتی نشان داده‌اند که عملکرد یک تکلیف حرکتی بعد از تمرین روان‌تر می‌شود و نوع تمرین مورد استفاده از اهمیت بالایی برخوردار است. هدف از انجام تحقیق حاضر، بررسی تأثیر تمرین با چالش ناپایداری تکلیف- ویژه بر عملکرد و اکتساب یک مهارت پرتابی بود. مواد و روش‌ها: طرح تحقیق حاضر نیمه تجربی و نوع آن کاربردی بود. 16 شرکت‌ کننده نیمه ماهر بسکتبال، تحت دو شرایط تمرینی با و بدون بازخورد بینایی قرار گرفتند. هر مرحله تمرینی شامل سه بلوک ده کوششی بود. قبل و بعد از مراحل تمرینی، عملکرد پرتابی افراد در دو مرحله پیش‌آزمون و پس‌آزمون بررسی شد. برای بررسی تفاوت افراد در مراحل تمرینی و آزمون از دو تحلیل واریانس با اندازه‌گیری‌های تکراری و t همبسته در سطح معنی‌داری 05/0 استفاده گردید. یافته‌ها: بین بلوک‌های تمرینی در هر دو مرحله تمرین تفاوت معنی‌دای وجود نداشت، اما در مرحله دوم تمرینی بین پیش‌آزمون و سایر بلوک‌ها و پس‌آزمون تفاوت معنی‌داری مشاهده شد (001/0 = P). به علاوه، بین پس‌آزمون‌های هر دو مرحله تمرینی تفاوت معنی‌داری دیده نشد. نتیجه‎گیری: ایجاد ناپایداری تکلیف- ویژه با حذف بازخورد بینایی، علاوه بر این که منجر به کسب مهارت پرتابی می‌شود، فرد را برای مقابله با شرایط ناپایدار حرکتی آماده می‌کند. به مربیان و درمانگران پیشنهاد می‌شود که برای آموزش مهارت‌های حرکتی و یا بازتوانی این مهارت‌ها، ورزشکاران و بیماران را تحت شرایط ناپایدار تمرین دهند؛ به طوری که آنان برای مقابله با این شرایط بهتر آماده شوند.

Title: بررسی درک هم‌معنایی واژگانی کودکان با کم‌توانی ذهنی آموزش‌پذیر در مقایسه با کودکان عادی

http://jrrs.mui.ac.ir/index.php/jrrs/article/view/2772

مقدمه: هم‌معنایی زمانی رخ می‌دهد که دو کلمه معنی مشابهی داشته باشد. از آن‌جایی که درک گفتار بر تولید آن مقدم است و با در نظر گرفتن این مسأله که کودکان با کم‌توانی ذهنی آموزش‌پذیر به دلیل مشکلات ذهنی و پایین بودن بهره هوشی در درک زبان با مشکلاتی روبه‌رو هستند، بنابراین انجام پژوهشی در زمینه چگونگی درک هم‌معنایی می‌تواند در امر ارتباط زبانی این کودکان مثمر ثمر واقع گردد. در نتیجه، هدف از انجام پژوهش حاضر، بررسی مقایسه درک هم‌معنایی با کودکان کم‌توانی ذهنی آموزش‌پذیر و کودکان عادی فارسی زبان بود. مواد و روش‌ها: روش مطالعه حاضر از نوع علی- مقایسه‌ای بود. داده‌های پژوهش از طریق آزمون محقق ساخته درک هم‌معنایی به دست آمد. بدین منظور، از 15 کودک با کم‌توانی ذهنی آموزش‌پذیر از مدارس استثنایی و 15 کودک عادی از مدارس عادی شهر جیرفت خواسته شد تا به این آزمون پاسخ دهند. به منظور تجزیه و تحلیل و همچنین، آزمون فرضیه پژوهش در درک هم‌معنایی، از آزمون Independent t و جهت مقایسه درک انواع هم‌معنایی از آزمون تحلیل واریانس چند متغیره استفاده گردید. یافته‌ها: نتیجه آزمون t Independent در ارتباط با درک هم‌معنایی در کودکان با کم‌توانی ذهنی آموزش‌پذیر نشان داد که تفاوت معنی‌داری بین دو گروه وجود داشت (05/0 > P). نتیجه‎گیری: کودکان با کم‌توانی ذهنی آموزش‌پذیر در درک هم‌معنایی نسبت به کودکان عادی با مشکلاتی مواجه هستند. با وجود این، کودکان با کم‌توانی ذهنی آموزش‌پذیر در درک هم‌معنایی دو کلمه متعلق به گویش‌های متفاوت و دو کلمه هم‌معنی متعلق به دو زبان متفاوت تفاوتی نشان ندادند.

Title: بررسی عوامل پیش‌بینی کننده ابتلا به استئوآرتریت زانو در سالمندان با تأکید بر فعالیت بدنی

http://jrrs.mui.ac.ir/index.php/jrrs/article/view/2804

مقدمه: طراحي برنامه‏هاي پيشگيري از استئوآرتریت زانو، مستلزم شناخت عوامل خطرزای آن است. هدف از انجام پژوهش حاضر، تعیین ارتباط متغیرهای سن، شاخص توده بدنی (Body mass index یا BMI)، تحصيلات، افسردگی، فعالیت بدنی و جنسیت با میزان خطر ابتلا به استئوآرتريت زانو در سالمندان بود. مواد و روش‌ها: 220 سالمند مبتلا به استئوآرتریت زانو و 220 سالمند سالم به روش نمونه‌گیری داوطلبانه انتخاب شدند و با استفاده از پرسش‌نامه‏ جمعیت‌شناختی، مقياس افسردگی سالمندان (Geriatric Depression Scale یا GDS) و پرسش‌نامه بین‌المللی فعالیت بدنی مورد بررسی قرار گرفتند. یافته‌ها: ارتباط همه متغیر‏ها با میزان خطر ابتلا به استئوآرتريت زانو، با استفاده از آنالیز رگرسیون لجستیک مدل پیش‌رونده و نسبت شانس بررسی شد. بین متغیرهای سن و جنسیت با خطر ابتلا به استئوآرتریت ارتباط معنی‌داری وجود داشت و زنان بیشتر از مردان در معرض خطر ابتلا به آسئوآرتریت بودند (050/0 > P). اضافه وزن و چاقی در سالمندان مبتلا به استئوآرتریت زانو بیشتر از سالمندان سالم مشاهده شد (001/0 > P). سطوح افسردگی ارتباط معنی‌داری را با خطر ابتلا به استئوآرتریت زانو نشان داد (005/0 > P). سطوح فعالیت بدنی با خطر ابتلا به استئوآرتریت زانو ارتباط معنی‌داری داشت و تعداد سالمندان غیر فعال مبتلا به استئوآرتریت زانو بیشتر از سالمندان سالم بود. هرچه سطح تحصیلات افزایش می‌یابد، احتمال ابتلا به استئوآرتریت زانو کمتر می‌شود و افراد با سطح تحصیلات پایین‏تر از دیپلم، شانس بیشتری برای مبتلا شدن به استئوآرتریت زانو داشتند (050/0 > P). نتیجه‎گیری: جنسیت، چاقی، افسردگی، عدم فعالیت بدنی، افزایش وزن، تحصیلات پایین‏تر از دیپلم و عدم بینش و آگاهی، از جمله عوامل خطر برای مستعد کردن افراد جامعه به استئوآرتریت زانو می‌باشد.

Title: ابزارهای کمی وکیفی در ابزارهای کمی و کیفی در مطالعات توانبخشی عضلات کف لگنتوانبخشی عضلات کف لگن

http://jrrs.mui.ac.ir/index.php/jrrs/article/view/2748

مقدمه: مشکلات بیومکانیکی عضلات کف لگن با توانبخشی ارزیابی و درمان می‌شود. ابزارهای ارزیابی و درمان مشکلات مجرای تحتانی ادرار علاوه بر ماهیت پاتالوژی بیماری، به فرهنگ بیمار نیز وابسته است. هدف از انجام مطالعه حاضر، معرفی ابزارهای ارزیابی و تحقیق مجرای تحتانی ادرار و کاربرد آن‌ها در مطالعات توانبخشی بود. مواد و روش‌ها: کلیه مطالعات بالینی که از اوایل سال ۲۰۰۰ تا پایان سال ۲۰۱۶ به زبان انگلیسی، مرتبط با تحقیق و درمان توانبخشی ثبت شده بود، با کلیدواژه‌های Pelvic floore dysfunction، Physical therapy، Rehabilitation، Urinary incontinence، Over active bladder، Pelvic floor muscle trainning، Lower urinary tract symptoms، Questionnaire و Instrument در پایگاه‌های اطلاعاتی معتبر جستجو شد. معیارهای ورود شامل مقالات از نوع کارآزمایی بالینی بود که به تحقیق و یا درمان توانبخشی مشکلات مجرای تحتانی ادراری پرداخته بود. مقالاتی که به زبانی غیر از انگلیسی نوشته شده بود، از مطالعه خارج گردید. سپس، منشأ هر ابزار جستجو و تعریف شد و در نهایت، تعداد هر ابزار جمع‌آوری، مورد تفکیک و ارزیابی قرار گرفت. یافته‌ها: تعداد ۱۸۱ مطالعه بین سال‌های ۲۰۰۰ تا ۲۰۱۶ به زبان انگلیسی چاپ شده بود. ۱۴۲ مطالعه مربوط به انواع بی‌اختیاری ادراری، جراحی پروستات و بیش‌فعالی مثانه هر کدام به ترتیب ۱۸ و ۱۴ مقاله استخراج شد. در ۷۱ درصد مطالعات حداقل یک پرسش‌نامه مربوط به کیفیت زندگی سؤال شده بود و در برخی از مطالعات پد تست و دیگر ابزارها به کار گرفته شد. نتیجه‎گیری: با وجودی که ارزیابی‌های کمی در تحقیقات توانبخشی به کار گرفته می‌شود، ولی ابزارهای پرسش‌نامه‌ای به خصوص پرسش‌نامه همراه ارزیابی کیفیت زندگی نقش مهمی در ارزیابی‌ها دارد.
